# Comparison of Sutureless *Versus* Suture Partial Nephrectomy for Clinical T1 Renal Cell Carcinoma: A Meta-Analysis of Retrospective Studies

**DOI:** 10.3389/fonc.2021.713645

**Published:** 2021-09-02

**Authors:** Wenjun Zhang, Bangwei Che, Shenghan Xu, Yi Mu, Jun He, Kaifa Tang

**Affiliations:** ^1^Department of Urology, Affiliated Hospital of Guizhou Medical University, Guiyang, China; ^2^Institute of Medical Science of Guizhou Medical University, Guiyang, China

**Keywords:** partial nephrectomy, renal cell carcinoma, meta-analysis, suture, sutureless

## Abstract

**Background:**

Partial nephrectomy (PN) is the recommended treatment for T1 renal cell carcinoma (RCC). Compared with suture PN, sutureless PN reduces the difficulty and time of operation, but the safety and feasibility have been controversial. This meta-analysis was conducted to compare the function and perioperative outcomes of suture and sutureless PN for T1 RCC.

**Methods:**

Systematic literature review was performed up to April 2021 using multiple databases to identify eligible comparative studies. According to the Preferred Reporting Items for Systematic Reviews and Meta-analysis (PRISMA) criteria, identification and selection of the studies were conducted. Meta-analysis was performed for studies comparing suture to sutureless PN for both T1a and T1b RCC. In addition, subgroup analysis was performed on operation time, warm ischemia time, estimated blood loss, and postoperative complications. Sensitivity analysis was used in analysis with high heterogeneity (operation time and estimated blood loss).

**Results:**

Eight retrospective studies were included with a total of 1,156 patients; of the 1,156 patients, 499 received sutureless PN and 707 received suture PN. The results showed that sutureless PN had shorter operative time (I^2^ = 0%, *P* < 0.001), warm ischemia time (I^2^ = 97.5%, *P* < 0.001), and lower clamping rate (I^2^ = 85.8%, *P* = 0.003), but estimated blood loss (I^2^ = 76.6%, *P* = 0.064) had no difference. In the comparison of perioperative outcomes, there was no significant difference in postoperative complications (I^2^ = 0%, *P* = 0.999), positive surgical margins (I^2^ = 0%, *P* = 0.356), postoperative estimated glomerular filtration rat (eGFR) (I^2^ = 0%, *P* = 0.656), and tumor recurrence (I^2^ = 0%, *P* = 0.531).

**Conclusions:**

In T1a RCC with low RENAL score, sutureless PN is a feasible choice, whereas it should not be overestimated in T1b RCC.

## Introduction

Partial nephrectomy (PN), in the surgical treatment of T1 renal cell carcinoma (RCC), is recommended according to the guidelines of the American Urology Association (AUA) and European Association of Urology (EAU) (1, 2). PN can provide better protection of renal function (3, 4), reduce the risk of severe cardiovascular events, and improve overall survival (5). However, the learning curve of PN is steep (6). In order to avoid the warm ischemic injury, the ischemic time usually limit to 25 min (7). Complete tumor resection, hemostasis, and suture in a limited time is a great challenge.

In recent years, some urologists have been focused on sutureless PN and tried to prove its safety and feasibility (2, 8–17). Without suture, the difficulty of operation is reduced, and the operation time is saved. However, the lack of reliable renal parenchyma suture may increase the potential risk of postoperative complications, which is controversial.

The superiority of PN in the surgical treatment of T1 RCC has been fully confirmed; by contrast, there is no meta-analysis comparing suture and sutureless PN. To fill this gap, we designed the present meta-analysis to compare the function and perioperative outcomes of the two techniques in PN for T1 RCC.

## Method

### Search Strategy

A literature search was performed in multiple databases (PubMed, Embase, and Web of Science) up to April 2021 to identify studies comparing suture to sutureless PN for T1 RCC. The diagnosis (kidney neoplasms, kidney cancer, renal carcinoma, renal tumor) and intervention (partial nephrectomy, nephron preservation and suture, surureless, and suture free) were used, respectively. We carried on the reference list search and citation literature retrieval for the full-text literature that met the research selection criteria.

### Inclusion Criteria, Study Eligibility, and Data Extraction

**Figure 1:**
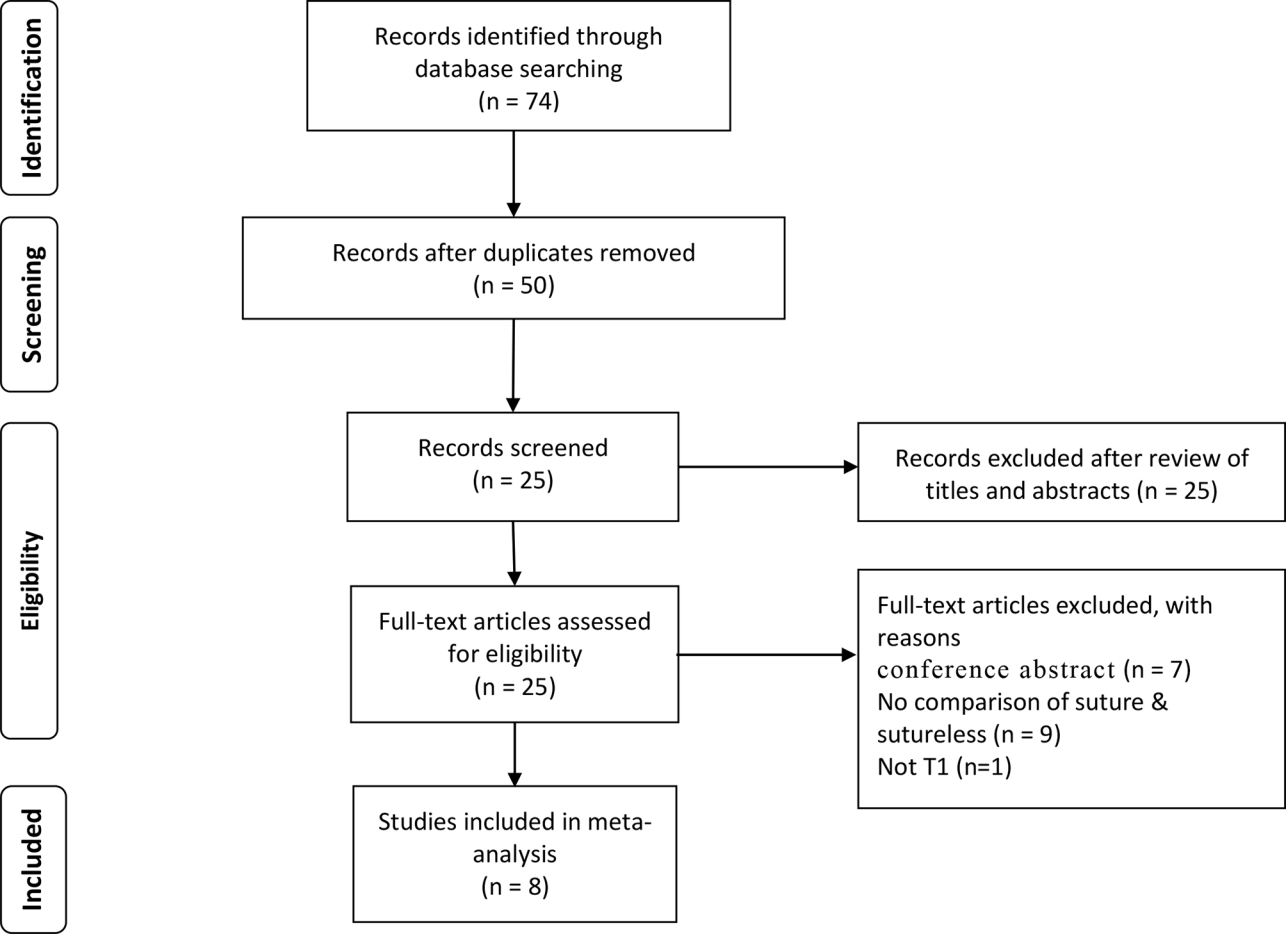
PRISMA flow chart of the study identification process.

The Preferred Reporting Items for Systematic Reviews and Meta-analysis (PRISMA) criteria were used for article selection (**Figure 1**), which was performed by two investigators (WZ and BC). The following study types were included: original studies comparing suture and sutureless PN were included regardless of the technique. All titles were screened for manuscripts written in the English language and only on adult patients. All studies were determined to include perioperative or functional outcomes. The titles of the articles were first reviewed to determine whether they might potentially fit the inclusion criteria. After assessing the abstract, a more comprehensive assessment was conducted by viewing the full text to determine whether the study should be included. Studies without primary data (i.e., case report reviews, commentaries, letters, and conference abstract) were excluded, but the reference lists were examined to identify that additional studies of interest had been included. References from the included studies were manually reviewed to identify additional studies of interest. Disagreement on whether or not an article should be included was resolved using a third reviewer (KT).

### Assessment of Study Quality

The quality of each study was determined using the Newcastle–Ottawa Scale (NOS) for nonrandomized controlled trials. The maximum score of the scale is 9. A total score of 5 or lower is considered low quality, 6–7 is considered intermediate quality, and 8–9 is considered high quality. Two researchers evaluated each study independently.

### Data Analysis

Data were extracted using a predefined data extraction form. Baseline demographics (age, T stage, RENAL Score, and baseline renal function), perioperative data (operative time, warm ischemia time, estimated blood loss, postoperative complications, surgical margins, and clamping rate), functional parameters [postoperative estimated glomerular filtration rat (eGFR)] and oncological outcome parameters (tumor recurrence) were extracted from the studies whenever available. For continuous outcomes, weighted mean difference (WMD) was used to measure differences; the risk ratio (RR) with 95% confidence interval (CI) was calculated for binary variables. For studies reporting medians and ranges [or interquartile ranges (IQR)], validated mathematical models were used to convert the median (range or IQR) to the mean [standard deviation (SD)] (18, 19). Between-study statistical heterogeneity was assessed using I^2^ and the Cochrane Q test. If there is no significant heterogeneity between the studies (*P* > 0.10, I^2^ < 50%), pooled estimates were calculated using a fixed-effects model; otherwise, using random effects model (*P* < 0.10, I^2^ > 50%). The significance level was set to α = 0.05, and 95%CI was taken. Begg’s and Egger’s tests were used to detect publication bias. Sensitivity analysis was used in high heterogeneity analysis to test the stability of the conclusion. This research used statistical software Stata12.0 to merge data.

## Results

This analysis included eight retrospective case–control studies with a total of 1,156 patients. All the included studies were compared the sutureless and suture techniques in laparoscopic PN for T1 tumors. Two of the studies used propensity score to match the preoperative baseline characteristics (**Table 1**). Subgroup analysis was performed between uncomplex (studies only included T1a RCC and RENAL score < 6) and complex subgroups (studies included T1b RCC and RENAL score ≥ 6), including operation time, warm ischemia time, estimated blood loss, and postoperative complications. Among the selected article, postoperative follow-up observation was carried out in six articles; only two articles reported recurrence. In addition, four articles compared postoperative eGFR. Due to different renal function evaluation criteria, we only selected two articles with the same criteria for meta-analysis. We analyzed the baseline characteristics according to tumor size, age, and preoperative renal function and evaluated selection bias to obtain more convincing conclusions (**Table 2**).

**Table 1:** Summary of baseline characteristics of the studies included in the meta-analysis.

Study	Study period	Study design	Study origin	T stage	Sutureless/suture			Surgical technique	SQ
					Cases (n)	RENAL score	FU (months)		
Feng Zhang (11)	2015–2018	RTP, MI, PSM	China	T1a–1b	116/116	6^a^/6^a^	6	Lap, Elec, Bio, RAC	7
Ching-Chia Li (14)	2015–2018	RTP, MI	China	T1a	33/19	5.7^a^/5.9^a^	29.3^a^/27.5^a^	sLap, Elec, Bio, RAC	7
Dachun Jin (10)	2014–2019	RTP, MI, PSM	China	T1a	65/189	5.3^a^/5.9^a^	22^a^	Lap, Elec, Bio, RAC	7
Daniele Tiscione (16)	2008–2009	RTP, MI	Italy	T1a	19/21	9.6^a^/9.4^a^	79.6	Lap, Bio, RAC	7
Jianfei Ye (10)	2012–2016	RTP, MI	China	T1a	78/126	4.8^a^/6.5^a^	47.2^a^/49.3^a^	Lap, Elec, Bio, RAC	6
Andrea Minervini (13)	2007–2010	RTP, MI	Italy	T1a	32/68	NA	NA	Lap, Elec, RAC	6
G. Hidas (17)	1993–2005	RTP, MI	Israel	T1a–1b	31/143	NA	NA	Lap, Elec, Bio, RAC	6
William. J 2005 (12)	1998–2004	RTP, MI	USA	T1a	75/25	NA	20.3^a^	Lap, Elec, Bio, RAC	7

Bio, biohemostatic material; Elec, electrocoagulation; FU, follow-up; Lap, laparoscopic; PSM, propensity score matching; RTP, retrospective; RAC, renal artery clamping; SC, single center; SQ, study quality according to the Newcastle–Ottawa scale; NA, not applicable. ^a^Median.

**Table 2:** Summary of baseline characteristics and outcomes of different analyses.

Outcomes	Included studies	Baseline, WMD or RR (*95% CI*)		
		Age	Tumor size	Preoperative eGFR, mL/min/m2
operative time, min	7	0.559 (−0.974, 2.091)	−0.190 (−0.543, 0.162)	NA
warm ischemia time, min	8	0.747 (−0.906, 2.400)	−0.223 (−0.525, 0.080)	NA
Ischemia rate, %	3	1.830 (−3.105, 6.765)	NA	NA
Estimated blood loss, ml	8	0.559 (−0.974, 2.091)	−0.223 (−0.525, 0.080)	NA
Postoperative complications	7	0.584 (−0.963, 2.131)	−0.223 (−0.525, 0.080)	NA
Preoperative eGFR ml/min/m^2^	2	3.638 (−3.018, 10.294)	0.200 (-0.193, 0.593)	−2.651 (−8.236, 2.933)
Positive surgical margin	4	1.529 (−0.575, 3.633)	−0.161 (−0.320, −0.002)	NA
Tumor recurrence	2	1.562 (−1.850, 4.975)	−0.200 (−0.375, −0.025)	NA

WMD, weighted mean difference; CI, confidence interval; eGFR, estimated glomerular filtration rat; NA, not applicable; RR, risk ratio.

**Figure 2:**
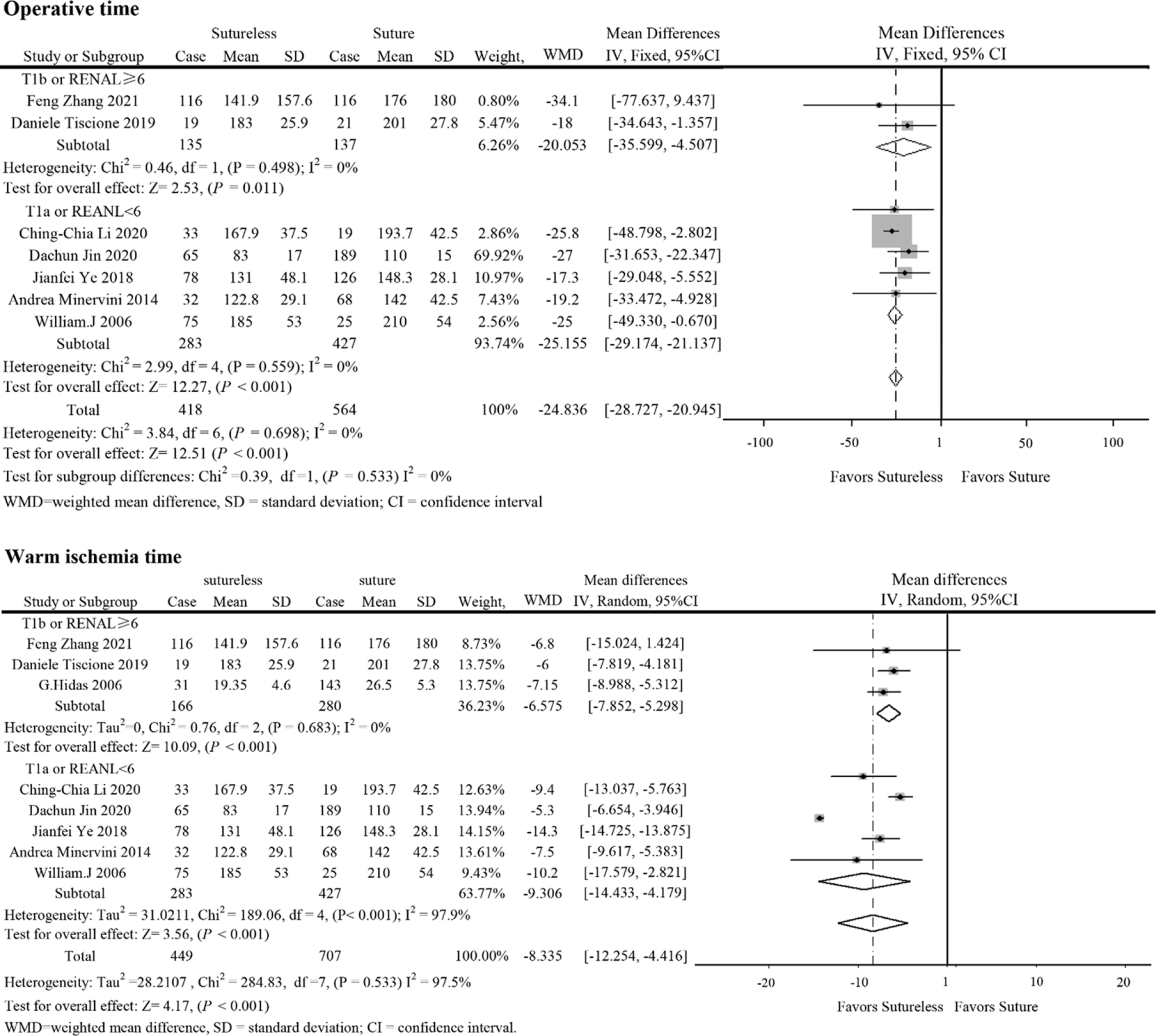
Forest plots of operative time and warm ischemia time for sutureless versus suture partial nephrectomy.

**Figure 3:**
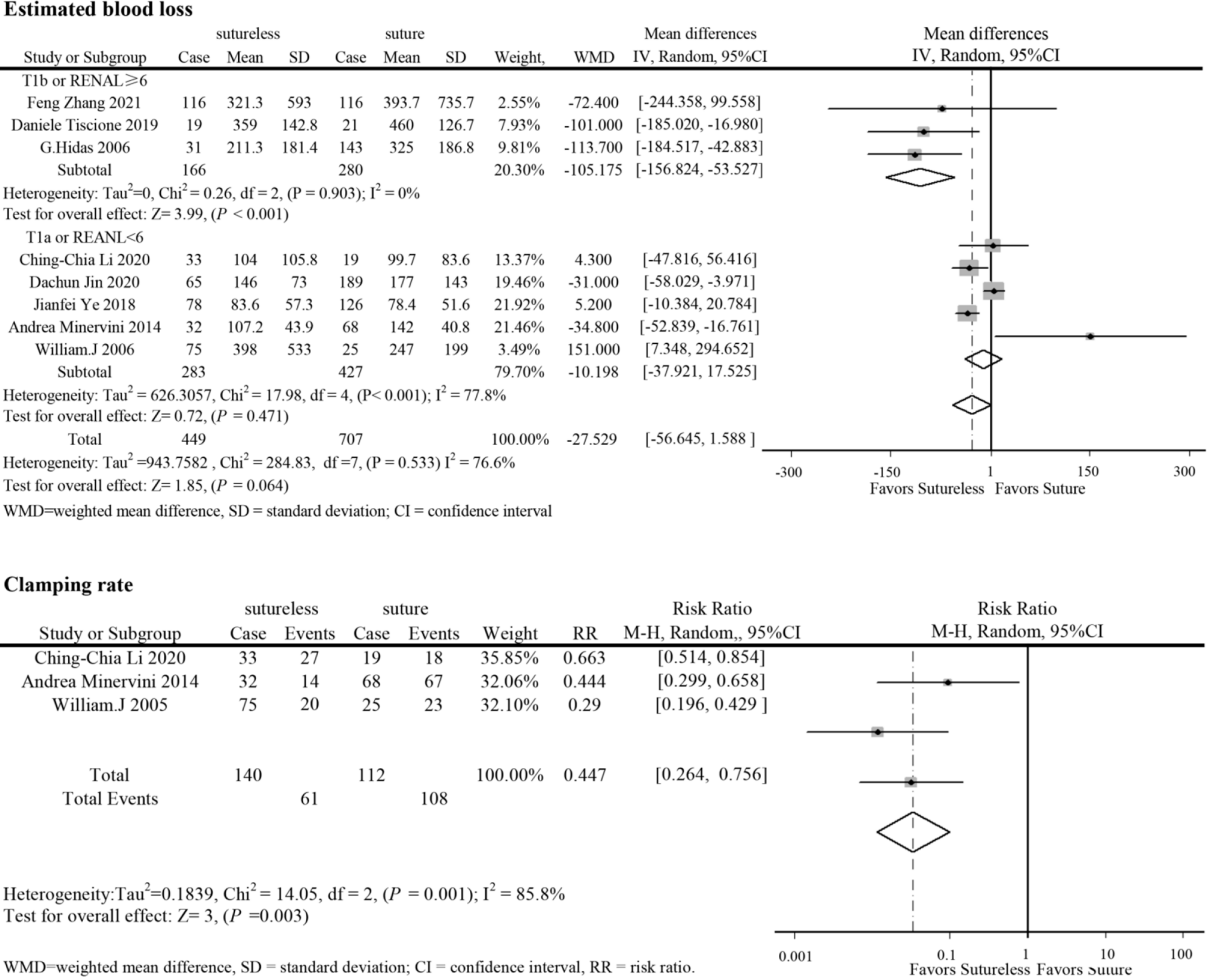
Forest plots of estimated blood loss and clamping rate for sutureless versus suture partial nephrectomy.

**Figure 4:**
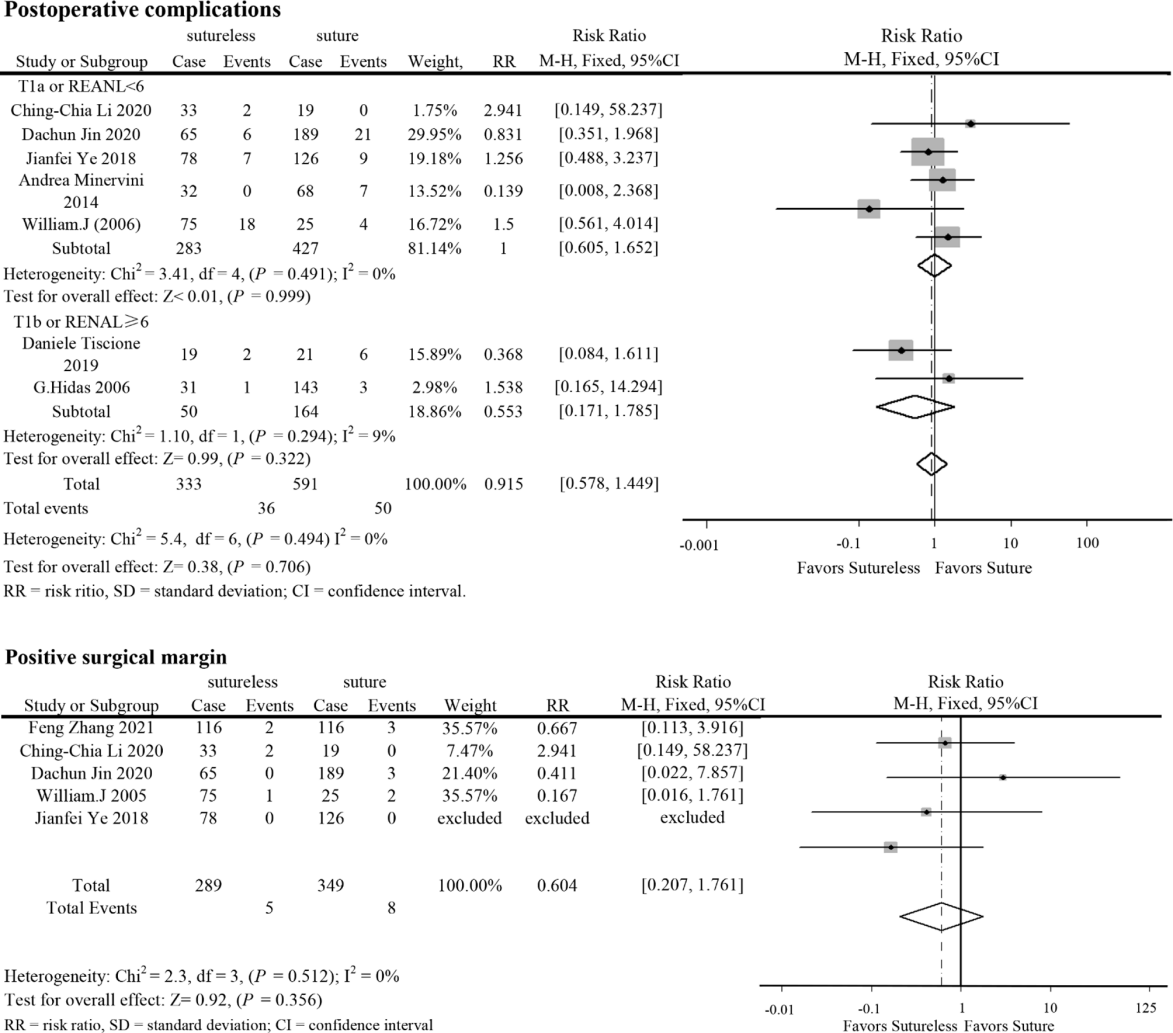
Forest plots of postoperative complications and positive surgical margin for sutureless versus suture partial nephrectomy.

**Figure 5:**
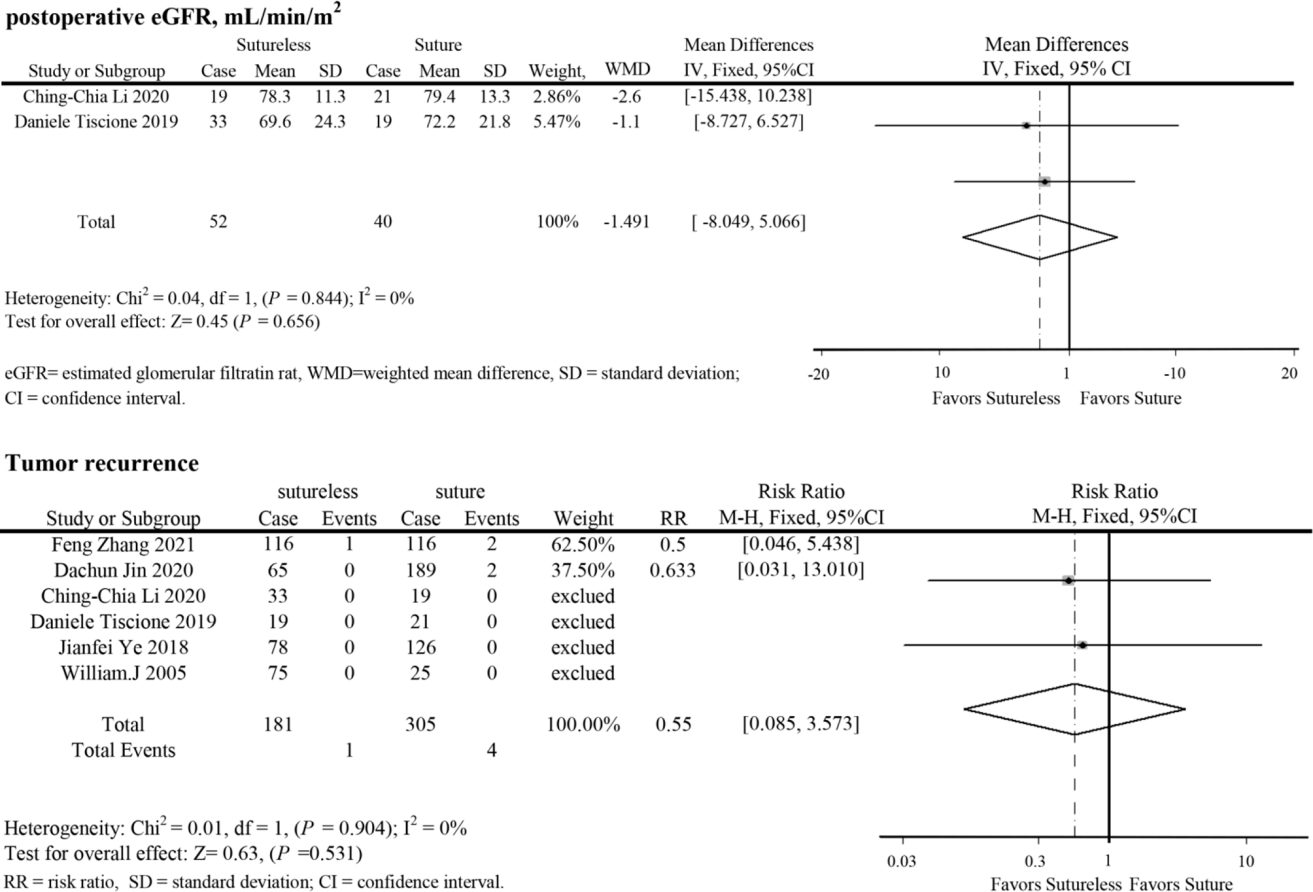
Forest plots of postoperative eGFR and tumor recurrence for sutureless versus suture partial nephrectomy.

In perioperative outcomes, the operation time was different (I^2^ = 0; WMD, −24.836; 95%CI, −28.727, −20.945; *P* < 0.001), and subgroup analysis showed that sutureless PN has shorter operation time than suture PN in uncomplex subgroup (I^2^ = 0; WMD, −20.053; 95%CI, −35.599 to −4.507; *P* = 0.011) and complex subgroup (I^2^ = 0; WMD, −25.155; 95%CI, −29.174 to −21.137; *P* < 0.001). Difference also existed in the warm ischemia time (I^2^ = 97.5; WMD, −8.335; 95%CI, −12.254 to −4.416; *P* < 0.001). In subgroup analysis, the ischemia time of sutureless PN was shorter than that of suture PN in the uncomplex subgroup (I^2^ = 97.9%; WMD, −6.575; 95%CI, −7.852 to −5.298; *P* < 0.001) and complex subgroup (I^2^ = 0; WMD, −9.306; 95%CI, −14.433 to −4.179; *P* < 0.001) (**Figure 2**). In addition, the rate of warm ischemia in the sutureless PN was lower than that in the suture PN (I^2^ = 85.8%; RR, 0.447; 95%CI, 0.264, 0.756; *P* = 0.003). There was no difference in estimated blood loss (I^2^ = 76.6%; WMD, −27.529; 95%CI, −56.645 to 1.588; *P* = 0.064); further subgroup analysis found sutureless PN had less blood loss in the complex subgroup (I^2^ = 0%; WMD, −105.175; 95%CI, −156.824 to −53.527; *P* < 0.001), while blood loss in the uncomplex subgroup had no difference (I^2^ = 77.8%; WMD, −10.198; 95%CI, −56.645 to 1.588; *P* = 0.064) (**Figure 3**). There was no significant difference in postoperative complications (I^2^ = 0; RR, 0.915; 95%CI, 0.578–1.449; *P* = 0.999), positive surgical margin (I^2^ = 0; RR, 0.604; 95%CI, 0.207–1.761; *P* = 0.356), postoperative renal function (I^2^ = 0; WMD, −1.491; 95%CI, −8.049 to 5.066; *P* = 0.656), and tumor recurrence (I^2^ = 0; RR, 0.55; 95%CI, −0.085 to 3.573; *P* = 0.531) (**Figures 4** and **5**).

Sensitivity analysis was used in analysis with high heterogeneity (operation time and estimated blood loss). After excluding the study of Jianfei Ye et al. (13), the heterogeneity of warm ischemia time decreased (I^2^ from 97.5% to 25.3%), but the results were consistent with previous studies. In addition, in comparing operation time, after excluding the study of Dachun Jin et al. (10), the results were also consistent with the previous studies.

Begg’s and Egger’s tests found no significant publication bias (**Table 3**).

**Table 3:** Publication bias.

Outcomes	Begg’s test	Egger’ s test	
		*P* value	95% CI
Operative time	*Pr* > |z| = 0.548	*P* > |t| = 0.317	[-.7072611, 1.784052]
Warm ischemia time	*Pr* > |z| = 0.174	*P* > |t| = 0.047	[0.1128378, 11.70039]
Clamping rate	*Pr* > |z| = 1.000	*P* > |t| = 0.774	[-2.500231, 2.650236]
Estimated blood loss	*Pr* > |z| = 1.000	*P* > |t| = 0.558	[-3.782394, 2.252696]
Postoperative complications	*Pr* > |z| = 0.368	*P* > |t| = 0.757	[-1.836992, 2.371768]
Positive surgical margin	*Pr* > |z| = 0.734	*P* > |t| = 0.554	[-7.953917, 11.07025]
postoperative eGFR	*Pr* > |z| = 1	NA	NA
Tumor recurrence	*Pr* > |z| = 1	NA	NA

eGFR, estimated glomerular filtration rat; CI, confidence interval, NA, not applicable.

## Discussion

At present, PN is the recommended method for surgical treatment of T1 tumors (1). A successful PN operation included surgical margin, slight decrease in renal function, and no serious postoperative complications (20). Maximum preservation of renal function is the original intention of PN (21). Saving the time of ischemia could reduce renal damage (22). In this study, sutureless PN had advantages in shortening the ischemia time and reducing operative blood loss. In addition, the rate of complete renal artery clamping was lower in the sutureless PN, which might have a potential advantage in the protection of postoperative renal function (23, 24).

Without reconstruction, sutureless PN could reduce the operation difficulty, which could reduce operation time. Although the lack of reconstruction might bring potential risks, in comparison of operative blood loss and postoperative complications, the application of biomaterials and electrocoagulation hemostasis in sutureless PN seems to have similar operative outcomes as suture PN. However, there was no difference in surgical blood loss between the two techniques in uncomplex subgroup, which reflected that reduction in clamping rate might lead to more blood loss. Due to the bias of retrospective study and the technical differences between each center that might directly affect the operative blood loss, operation time, and ischemia time, it cannot fully prove that sutureless PN has more advantages. In addition, with the continuous optimization and maturity of suture PN, the difference in operative blood loss and ischemia time might be reduced (25).

Segmental renal artery ligation caused by the suture might lead to the loss of functional renal parenchyma (26–28). Sutureless PN seems to reduce the potential impact on postoperative renal function, but no difference in postoperative eGFR was found in our study. In the study of Zhang et al. and Jin et al., it was shown that the decline in renal function after sutureless PN was lower than that after suture PN (10, 11). Due to the different diagnostic criteria in the included studies, we could not evaluate the difference in renal function decline to prove that sutureless technique is better than suture technique in renal function protection. In this study, the low positive surgical margin and tumor recurrence reported in the included studies might benefit from the resection near the pseudocapsule of renal tumor, which had been proven to be safe for oncology outcomes (29, 30). Due to the incomplete follow-up data in the included studies, more detailed data are needed to assess the long-term prognosis of sutureless and suture PN.

In this study, outcomes of sutureless and suture PN in perioperative were similar, and sutureless PN might have potential advantages in protecting renal function. However, in larger and more complex T1b RCC, which requires more extensive parenchyma resection and reconstruction, the potential risk may be greater. Overall, under strict preoperative and intraoperative evaluation, sutureless PN could be a feasible choice for smaller T1a RCC, whereas should not be overestimated in T1b RCC.

Our research has some limitations. First, the meta-analysis of retrospective studies, which have an inherent bias, could not fully compare the pros and cons between the two techniques. Second, the technical differences and diagnostic criteria between different time periods and different centers and heterogeneity between studies were inevitable. Analysis of the sources of heterogeneity requires more detailed subgroup data. Third, due to the lack of oncology outcome-related data, we cannot better evaluate the surgical prognosis. Lastly, only eight retrospective studies are included in this analysis, and more high-quality studies are needed in the future.

## Conclusion

Our meta-analysis suggested that the two surgical techniques have similar perioperative outcomes in T1a RCC with low RENAL score. In addition, sutureless PN might have potential advantages in the protection of renal function. In some cases, sutureless PN is a feasible choice under strict preoperative and intraoperative evaluation. However, application in larger and more complex T1b needs to be cautiously made. More well-designed prospective randomized clinical trials are needed for further research in the future.

## Data Availability Statement

The original contributions presented in the study are included in the article/supplementary material. Further inquiries can be directed to the corresponding author.

## Author Contributions

Study concept and design: KT and WZ. Data acquisition: WZ, BC, SX, YM, and JH. Data analysis: WZ, BC, and SX. Drafting of manuscript: WZ. Critical revision of the manuscript: KT. All authors contributed to the article and approved the submitted version.

## Funding

This study was supported by the Science and Technology Fund Project of Guizhou Health Commission (gzwkj2021-211).

## Conflict of Interest

The authors declare that the research was conducted in the absence of any commercial or financial relationships that could be construed as a potential conflict of interest.

## Publisher’s Note

All claims expressed in this article are solely those of the authors and do not necessarily represent those of their affiliated organizations, or those of the publisher, the editors and the reviewers. Any product that may be evaluated in this article, or claim that may be made by its manufacturer, is not guaranteed or endorsed by the publisher.
